# Investigation of job satisfaction amongst voluntary, counseling and testing centers and health centers in Iran

**DOI:** 10.1186/s40359-022-00972-9

**Published:** 2022-11-08

**Authors:** Mohammadreza Heydari, Marjan Faghih, Yasaman Karimzadeh, Hassan Joulaei, Fariba Qhiasi, Negin Dadmanesh, SeyedAhmad SeyedAlinaghi, Farzane Hosseini, Soloman Yeilaghi, Mohammad Reza Miri, Farzane Pirmadah, Wali Amini, Marjan Meshkati, Parvin Afsar Kazerooni, Nasim Nasiri Moghadam, Zahra Heydari, Morteza Mehraeen

**Affiliations:** 1 HIV/AIDS Research Center, Institute of Health, Shiraz University of Medical Sciences, Shiraz, Iran; 2 Department of Biostatistics, School of Medicine, Arak University of Medical Sciences, Arak, Iran; 3 Health Policy Research Center, Institute of Health, Shiraz University of Medical Sciences, Shiraz, Iran; 4 Tehran University of Medical Sciences (TUMS), Iranian Research Center for HIV/AIDS (IRCHA), Tehran, Iran; 5 Voluntary Counselling and Testing Center, Health Deputy, Birjand University of Medical Sciences, Birjand, Iran; 6 Birjand, Iran; 7 The Persian Gulf Marine Biotechnology Research Center, The Persian Gulf Biomedical Sciences Research Institute, Bushehr University of Medical Sciences, Bushehr, Iran; 8 Shahid Sadoughi university of Medical Sciences, International Campus, Yazd, Iran; 9 Kurdistan University of Medical Sciences, Sanandaj, Iran; 10 Isfahan University of Medical Sciences, Isfahan, Iran; 11 Center for Communicable Disease Control, Ministry of Health and Medical Education, Tehran, Iran; 12 Kerman University of Medical Sciences, Kerman, Iran; 13 Department of Biology, Shiraz University of Sciences, Shiraz, Iran; 14 Voluntary Counseling and Testing Center, 2nd floor, Lavan Ave, Delavaran-e Basij Blvd, Khatoun Sq, Shiraz, Fars Iran

**Keywords:** Job satisfaction, Healthcare personnel, HIV

## Abstract

**Background:**

Job satisfaction is the persons’ feeling about their job and if personnel have not good feel to his work, can destroy all plans, intentionally or unintentionally. The present research aims to investigate and compare job satisfaction in the employees and therapists of Voluntary, Counseling and Testing Centers versus Health centers in 9 provinces of Iran.

**Methods:**

All employees of Voluntary, Counseling and Testing Centers were included from Fars, Bushehr, Tehran, Isfahan, South Khorasan, Kurdistan, Kermanshah, Kerman, and Yazd provinces as case group and 103 staffs of similar Health centers selected with the same ratio as the staffs of Voluntary, Counseling and Testing Centers as control samples and answered to Minnesota Satisfaction Questionnaire (MSQ).

**Results:**

50.5% of Health centers employees and 54% of Voluntary, Counseling and Testing Centers employees had high job satisfaction. The highest satisfaction levels were reported in Fars and Kurdistan provinces and the lowest satisfaction levels were reported in Kermanshah and Bushehr.

**Conclusion:**

According to the findings, in the Iranian treatment centers, the employees’ satisfaction were at the same level regardless of their position and workplace. Also, the eastern and western regions of the country reported higher satisfaction score than the southern and central regions.

## Background

Job refers to an activity or a set of activities performed during a specific period (for example, 8 h a day) for a specific cost [1]. Studies have shown that people with a common job and adequate income are more secure against psychological disorders and especially depression [2]. Also, it has been found that depending on their job satisfaction, employed people can have a higher level of efficiency and mental health [3]. However, as a job can make a person secure against psychological disorders, it can make him/her vulnerable, too; particularly, when the person’s activity is free of mobility, creativity, social and professional associations, variety, or even with excessive mobility, high pressure, and complicated skills [4]. If the job’s atmosphere imposes mental pressure to individuals in long term, they will be more probable of being dissatisfied and unwilling to do work.

Job satisfaction is the persons’ feeling about their job [5]. Therefore, dissatisfaction means an unfavorable feeling, continuous sadness, excessive tiredness, a sense of incompetency and etc. Job dissatisfaction increased cortisol level in blood and consequently leads stress and anxiety [6]. High cortisol levels in long term can cause depression, psychosomatic symptoms, cardiovascular disorders, digestive disorders, migraine and etc. that most of them are considered conversion disorders [7]. Job dissatisfaction can also affect family space, friendship, and non-organizational cooperation ; So, it can be expanded to various situations [8].

People such as emergency staff and firefighters who have risky jobs, are more prone to job dissatisfaction. On the other hand, dissatisfaction can vary even in different parts of a system. According to Tavakoli et al., 61.1% of the Iranian professional nurses reported a medium level of satisfaction, 22.2% of them reported a low level of satisfaction, and 16.7% reported a high level of satisfaction [9]. Nourani Sadodin et al. showed that mean scor of job satisfaction in midwives in Mashhad is 44.85 ± 8. The majority of midwives (59.0%) were satisfied from their job and most of them (61.5%) had moderate level of job stress [10].

Zorec, Rusac, and Ogresta found that the mental healthcare staff report different levels of job satisfaction. However, their satisfaction level was not related to their job [11]. Wright and Bonett reported that job satisfaction has the strongest (negative) association with job change [12]. Zaid Al-Hamdan et al. reported a positive association between the nurses’ job satisfaction and their workplace, so that the nurses who working in public hospitals, reported higher levels of satisfaction compared to the nurses who working in educational hospitals. So, there was a positive association between the nurses’ workplace and their intention to stay [13]. Pineau et al. reported a significant association between structural empowerment and perceived staffing adequacy with job satisfaction [14]. However, Vlachos and Panagopoulos and Rapp showed that in corporate social responsibilities (CSRs), if employees recognize their managers as charismatic person, they will have higher job satisfaction and motivation. Charisma also creates a sense of job satisfaction in leaders [15]. However, in their systematic review, Kuoppala et al. reported that management style, the number of sick leaves, and even disability has no association with job satisfaction [16].

Voluntary counseling and testing centers are places for specialized treatment and counseling of HIV patients. These centers have usually few, but high-risk patients. Most of the patients referring to these centers are addicts, sex workers, and antisocial people. So, this environment can endanger the health of the employees and therapists who working in these centers. All treatments and services like midwifery services, psychological and psychiatric treatment, social work, education of the disease and etc. is accessible and free of charge; Also for HIV patients, this place is safe and without stigma.

On the other hand, health centers (HC) have a lot of patients with different emergency or non-emergency conditions but despite the infection risk, the physician and employees are exposed to lower safety threats. According to the aforementioned conditions, the question is that, which of the centers’ employees have more job satisfaction? It seems that no research has been done to examine the satisfaction status of the VCTs and compare their situation with HCs. Therefore, the present research aims to investigate and compare job satisfaction in the employees and therapists who working in VCT and HCs in 9 provinces of Iran.

## Methods

This research is a cross-sectional study simultaneously performed in 9 provinces of Iran. This study approved by Shiraz University of medical sciences with the ethical code: IR.SUMS.REC.1398.865.

It should be mentioned that VCTs only exist in the center of provinces. So, the statistics are only obtained from the province centers. All people who worked as employees or therapists in VCT were included in the study as group1. Attempts were made to select the group 2 of the same number of subjects with the same positions that working in HCs. With this approach, the number of participants in health centers, increased slightly and due to the small sample size, we preferred not to eliminate additional participants.

*Inclusion criteria* all of employees (physician, personnel, Service force, chief and etc.) in VCTs and all personnel that had the VCT’s employees’ characteristics.

*Exclusion criteria* everyone who did not want to participate in the study and people who were employed in VCTs but did not have similar personnel or position in HCs.

As presented in the Table 1; Fars and Tehran had the largest number of employees. Minnesota Satisfaction Questionnaire and its guide were sent to the researcher representatives in each province. The research was performed from January 2020 until March 2020 with cooperation of 9 co-researchers in 9 provinces.

**Table 1:** Number of participants

Province	VCTs			HCs		
	No. of non-therapist staff	No. of therapists	Total	No. of non-therapist staff	No. of therapists	Total
Fars	20	5	25	20	5	25
Bushehr	3	1	4	5	0	5
Isfahan	11	1	12	10	3	13
Kerman	5	3	8	4	2	6
Kermanshah	10	3	13	6	1	7
Tehran	14	3	17	24	4	28
South Khorasan	2	1	3	2	1	3
Kurdistan	6	2	8	10	2	12
Yazd	2	1	3	3	1	4
total	73	20	94	84	19	103

The used Minnesota Satisfaction Questionnaire (MSQ) has 19 items that are scored based on Likert scale including the choices of “quite disagree, disagree, no idea, agree, and quite agree”. This questionnaire measures the 6 subscales include of payment system (3 items), the type of job (3 items), achievement opportunities (33 items), the organizational atmosphere (2 items), leadership style (4 items), and the physical conditions (3 items) [17]. In this questionnaire, the scores of 19–38 indicate low levels of job satisfaction, 38–57 indicate medium levels of job satisfaction, and above 57 indicate high levels of job satisfaction. The reliability and validity of this questionnaire have been obtained equal to 0.86 in an Iranian population.

The collected data were analyzed by SPSS 23. Means comparisons between groups were done by independent T-test. One-way analysis of variance (ANOVA) and Tukey post hoc test were used to compare a factor between three or more groups. The association between two classified factors was studied by Chi-square and Fisher’s exact test. The correlation between quantitative factors such as age and the score of satisfaction was studied by Spearman’s correlation coefficient test. The normality of the factors was checked by the Shapiro-Wilk test. The basic significance level was considered 0.05 in all the tests.

## Results

Group size of health centers was 103 persons and Group size of Voluntary, Counseling and Testing Centers was 94 persons. Mean and SD of age in general was 39.97 ± 9.02 and respectively in HCs and VCTs was 38.43 ± 9.64 and 41.56 ± 7.99. Work experience in HCs and VDTs were 56.52 ± 63.73 months (about 5years) and 94.01 ± 65.86 months respectively (about 8 years).

As presented in the Table 2; there was a significant relationship between sex, age, work time, work experience and second job with each group (HC, VCT).

**Table 2:** Demographic characteristics

Factors	HC (n = 103)	VCT (n = 94)	Statistic	df	*p* value
*Sex*					
Men	25 (24.27)	41 (43.61)	8.26^a^	1	0.004
Women	78 (75.27)	53 (56.38)			
*Age (years)*					
20–30	28 (27.2)	7 (7.4)	14.59 ^a^	3	0.002
31–40	26 (25.24)	37 (39.4)			
41–50	37 (35.92)	36 (38.3)			
> 50	11 (10.67)	14 (14.9)			
*Marital status*					
Single	28 (27.18)	15 (16)	3.88 ^a^	2	0.144
Married	73 (70.9)	72 (79.8)			
Divorce	2 (2)	4 (4.2)			
Education					
*Primary education*	9 (8.7)	8 (8.5)	4.88 ^a^	4	0.3
Associate	5 (4.9)	7 (7.4)			
Bachelor	57 (55 0.3)	39 (41.5)			
Master	13 (12.6)	20 (21.3)			
Physician	19 (18.5)	20 (21.3)			
*Employment status*					
Official	46 (44.6)	51 (54.3)	4.99^b^	–	0.26
Contractual	23 (22.3)	18 (19.1)			
Apprenticeship	8 (7.8)	2 (2.1)			
Contracting	25 (24.3)	23 (24.5)			
Others	1 (1)	0(0)			
*Position*					
Physician	19 (18.4)	20 (21.3)	0.29 ^a^	1	0.59
Personnel	84 (81.6)	73 (77.7)			
*Work time*					
Part time	13 (12.6)	22 (23.4)	3.97 ^a^	1	0.046
Full time	87 (84.5)	69 (73.4)			
*Work experience (month)*					
1–72	79 (76.7)	44 (46.8)	27.97 ^b^	4	*p* < 0.001
73–144	15 (14.6)	26 (27.7)			
145–216	2 (1.9)	18 (19.1)			
217–288	2(1.9)	2(2.1)			
289–360	2 (1.9)	0 (0)			
*Second job*					
Yes	14 (13.6)	25 (6.6)	5.26 ^a^	1	0.022
No	88 (85.4)	68 (72.3)			

*HC* health centers,* VCT* voluntary, counseling and testing centers

^a^Chi-square test

^b^Fisher’s exact test

**Table 3:** The Investigation and association between three levels of satisfaction and the health center type

Center	Minnesota score					
	19–38 N (%)	39–57 N (%)	58–90 N (%)	Total N (%)	χ^2^ (df)	(*p* value)
HC	8(7.8)	43(41.7)	52(50.5)	103(100)	2.35(2)	(0.31)
VCT	3(3.2)	37(39.4)	54(57.4)	94(100)		

As presented in the Table 3; there was no significant association between the satisfaction score and working in the two centers.

The mean of satisfaction score in HC and VCT centers were 57 ± 13.56 and 58.21 ± 10.38 respectively.

The results showed no significant difference between the mean score of satisfaction and its subscale with the service type, period factors and participants employment status. There was only a significant negative correlation between age and management; so higher age was associated with a decreased management score (*p* = 0.027, r= -0.158).

Finally, there was no significant difference between the satisfaction score of men and women.

According to Table 4, results showed no significant difference in any of the subscales between two treatment centers. The results showed that in HCs, the highest and lowest satisfaction scores in the subscale of achievement opportunity were respectively reported in South Khorasan and Bushehr provinces [F (8): 3.06, *p* = 0.004]. In the physical condition subscale, the highest and lowest scores were respectively reported in South Khorasan and Kermanshah with a significant difference between the two provinces [F (8): 3.03, *p* = 0.005].

**Table 4:** Satisfaction score and its dimensions; a comparison between two centers

Corresponding subscales	Center	Mean	SD	Student’s t-test	*p* value
Payment system	HC	6.37	3.35	0.35	0.73
	VCT	6.52	2.66		
The type of job	HC	14.24	3.26	0.64	0.53
	VCT	14.52	2.86		
Achievement opportunity	HC	7.61	3.16	1.59	0.11
	VCT	8.31	2.97		
Work atmosphere	HC	7.09	1.96	1.42	0.16
	VCT	7.48	1.9		
Management style	HC	12.45	2.54	0.12	0.91
	VCT	12.5	2.59		
Physical conditions	HC	9.23	3.48	0.7	0.48
	VCT	8.88	3.49		
The total score of satisfaction	HC	57	13.56	0.7	0.49
	VCT	58.21	10.38		

In VCTs, the highest and lowest satisfaction scores in the payment system subscale were respectively reported in Kerman and Yazd [F (8): 2.98, *p* = 0.005]. In the subscale of achievement opportunity, the highest and lowest scores were respectively reported in Kerman and Tehran [F (8): 1.06, *p* < 0.001]. In the work atmosphere subscale, the highest and lowest scores were respectively reported in Bushehr and Yazd with a significant difference [F (8): 6.67, *p* < 0.001]. In the physical condition subscale, the highest and lowest scores were respectively reported in Kerman and Isfahan with a significant difference [F (8): 3.72, *p* = 0.001].

The provinces were classified into four regions including the central provinces (Isfahan and Tehran), eastern provinces (Kerman, South Khorasan, and Yazd), western provinces (Kurdistan and Kermanshah), and southern provinces (Fars and Bushehr). Classifying the provinces into 4 groups showed that there was no significant difference between the provinces in terms of satisfaction in HCs [F (3):1.86, *p* = 0.142].

In VCTs, the eastern provinces reported higher satisfaction levels (65.67 ± 11.11) than the central provinces [52.69 ± 8.23, F (3): 6.56, *p* < 0.001]

According to the results of the post hoc test, there was a difference between the central and western provinces (mean difference (std.Error):12.98(3.04), *p* < 0.001) also the central and eastern provinces (mean difference (std.Error): 7.45(2.74), *p* = 0.038).

As seen in Table 5, there is a significant difference between the HCs in terms of job satisfaction level; the highest satisfaction levels were reported in Fars and Kurdistan provinces and the lowest satisfaction levels were reported in Kermanshah and Bushehr.

**Table 5:** Investigation and Comparison of the total mean scores of satisfaction in each center of the provinces

Center	Province	Participants	Mean	SD	Statistical Index	*p* value
HCs	Fars	25	64.96	11.82	18.4	0.018
	Kurdistan	12	59.92	18.88		
	Kermanshah	6	49.67	7.92		
	Kerman	6	56.17	16.64		
	Yazd	4	49.75	12.18		
	Isfahan	13	55.15	8.17		
	South Khorasan	3	63	4.58		
	Tehran	28	53.89	12.87		
	Bushehr	5	45.8	13.14		
VCTs	Fars	25	56.84	10.64	26.64	0.001
	Kurdistan	8	58.15	3.58		
	Kermanshah	13	61.15	8.56		
	Kerman	9	71	9.76		
	Yazd	3	53	5.19		
	Isfahan	12	53	9.07		
	South Khorasan	3	62.33	8.38		
	Tehran	17	52.47	8.05		
	Bushehr	4	68.75	10.78		

In VCTs, the highest satisfaction levels were reported in Kerman and Bushehr provinces and the lowest satisfaction levels were reported in Tehran, Isfahan, and Yazd provinces (*p* = 0. 001).

## Discussion

The present research was aimed to investigate and compare job satisfaction in Health centers (HC) and Voluntary, Counseling and Testing Centers (VCT) in 9 provinces of Iran. According to the results, most of the employees were middle-aged (about 39 years) and their work experience was between five months until thirty years.

Although it was expected to find a significant difference between the satisfaction level in HCs and VCTs, the results included in Table 1 showed that about 50% of the therapist and employees working in both centers reported high levels of satisfaction and 45% of them reported medium levels of satisfaction. These findings is according with the results reported by Tavakoli et al. and Nourani Sadodin et al. [9, 10]. So, this lack of difference may be due to the fact that, although there are noVCT’s risks in HCs, but the overcrowding of patients and the risk of other infectious diseases remain. This research showed no significant association between satisfaction and education level. This finding is contradictory to the results reported by Nourani Sadodin et al. [10]. Meanwhile, the satisfaction of the employees and therapists of the two centers had no significant relationship with working hours, type of service and work time. These findings are also contradictory to the results reported by Al-Hamdan et al. [13]. This difference can be caused by the nature of hospital work and work in these centers.

Present research showed no significant association between recruitment type, education, work type and satisfaction. while Ogresta, Rusac, and Zorec and Bonett and Wright, reported that the payment system and financial affairs are directly effective in the employees’ satisfaction [11, 12]. This difference can be related to the Iranians’ psychological attributes. Perhaps they are resilience; and medical jobs are sacred for them. However, the mentioned results are related to 9 provinces, and separating the provinces leads to different results. The results showed no significant difference between job satisfaction and gender in Iran. Since findings showed higher level of job satisfaction for women in USA than men [18] and lower for men in china[19]. Certainly this dispute in finding related to difference in social factors between three countries. The difference in the salaries received by men and women, as well as the difference in their ability to endure hardships, can be one of the causes of these differences in different countries. But in Iran, both genders are exposed to the same occupational pressure, benefits and harms.

The level of job satisfaction had a significant relationship only with age and management style. The results showed increasing in employees ‘’age’’ has decreased their satisfaction from management status. In other words, by increasing their work experience, employees can evaluate the competencies and capabilities of managers and cannot accept less experienced managers. This finding is contradictory to the result reported by Vlachos and Panagopoulos and Rapp. They suggested that whatever a manager is more charismatic, employees will be more satisfied. In this regard, the individuals’ experience and background determine the satisfaction level [15].

Based on the data in Tables 2 and 3, the job satisfaction in HCs and VCTs in eastern and southern provinces is at a higher level than other provinces. This difference can be due to the difference in the management of the centers, the number of patients visiting the centers, or the cultural differences of the residents of these provinces. It is necessary to understand this issue by designing a research based on the etiology of the difference in job satisfaction in the treatment staff of these centers.

## Limitation

This study has several limitations. There was only one VCT in the center of each province, and the composition of its employees was also fixed, and it might be that there was no exact control sample in some centers. Second, Due to the lack of staff in VCTs, it was very difficult to homogenize the HC sample and third, Some of VCT’s staffs did not cooperate and there was no replacement for them.so further research will be need with larger sample size and more dispersion are needed.

## Conclusion

This research suggested that in the questioned treatment centers, the employees’ satisfaction will be at the same level regardless of their position and workplace. Meanwhile, the eastern and western regions of the country reported a higher level of satisfaction compared to the southern and central regions. However, the employees’ dissatisfaction with management styles suggests the necessity of assigning the experienced employees as the managers of health centers. The difference between the satisfaction levels in the studied provinces can make this question: Is the current situation the result of differences in the culture of the regions or the lack of work justice in medical centers?

## Data Availability

The datasets during and/or analysed during the current study available from the corresponding author on reasonable request.

## Abbreviations

HIV: Human immunodeficiency virus
HC: Health Centre
VCT: Voluntary, Counseling and Testing Centers
MSQ: Minnesota Satisfaction Questionnaire
